# Effects of fecal microbiota transplantation on metabolic health of DBA mice

**DOI:** 10.3389/fmicb.2024.1352555

**Published:** 2024-02-20

**Authors:** Wenxin Ye, Jinghui Fan, Wenzi Wu, Zhuo Chen, Qixin Huang, Lichun Qian

**Affiliations:** ^1^Key Laboratory of Animal Nutrition and Feed Science in East China, Ministry of Agriculture, College of Animal Sciences, Zhejiang University, Hangzhou, China; ^2^Hangzhou Academy of Agricultural Sciences, Hangzhou, China; ^3^Hainan Institute of Zhejiang University, Sanya, China

**Keywords:** DBA mice, C57BL/6 mice, fecal microbiota transplantation, transcriptome analysis, 16S RNA sequencing, Wistar rats

## Abstract

**Introduction:**

Numerous studies have demonstrated that C57BL/6 mice exhibit superior growth rates and overall growth performance compared to DBA mice. To investigate whether this discrepancy in growth performance is linked to the composition of gut microorganisms, we conducted fecal microbiome transplantation (FMT) experiments.

**Methods:**

Specifically, we transplanted fecal fluids from adult C57BL/6 mice, high-fat C57BL/6 mice, and Wistar rats into weaned DBA mice (0.2mL/d), and subsequently analyzed their gut contents and gene expression through 16S rRNA sequencing and transcriptome sequencing. During the test period, C57BL/6 mice and Wistar rats were provided with a normal diet, and high-fat C57BL/6 mice were provided with a high-fat diet.

**Results:**

The results of our study revealed that mice receiving FMT from all three donor groups exhibited significantly higher daily weight gain and serum triglyceride (TG) levels compared to mice of CK group. 16S rRNA sequensing unveiled substantial differences in the abundance and function of the gut microbiota between the FMT groups and the CK group. Transcriptome analysis revealed a total of 988 differential genes, consisting of 759 up-regulated genes and 187 down-regulated genes, between the three experimental groups and the CK group. Functional Gene Ontology (GO) annotation suggested that these genes were primarily linked to lipid metabolism, coagulation, and immunity. Pearson correlation analysis was performed on the differential genes and clusters, and it revealed significant correlations, mainly related to processes such as fatty acid metabolism, fat digestion and absorption, and cholesterol metabolism.

**Discussion:**

In summary, FMT from dominant strains improved the growth performance of DBA mice, including body weight gain, institutional growth, and immune performance. This change may be due to the increase of probiotic content in the intestinal tract by FMT and subsequent alteration of intestinal gene expression. However, the effects of cross-species fecal transplantation on the intestinal flora and gene expression of recipient mice were not significant.

## 1 Introduction

In recent years, there has been a significant focus on understanding the impact of gut microbes on the performance of the host organism. In mammals, the gut microbiota plays a pivotal role in regulating lipid metabolism, primarily through the influence of compounds like short-chain fatty acids, secondary bile acids, trimethylamine, and proinflammatory agents derived from bacteria, such as lipopolysaccharides (Schoeler and Caesar, 2019). This intricate process predominantly centers around the bile acid signal transduction pathway, with a heavy reliance on key receptors like the farnesoid X receptor (FXR), the G-protein-coupled bile acid receptor (Gpbar/TGR5), the short-chain fatty acid signaling pathway, and the functioning of enteroendocrine cells (Yu et al., 2019). FMT is a method of transplanting bacteria from the feces of a pre-selected donor into the intestines of a recipient in order to restore the homeostasis of the recipient’s intestinal flora, and thus restore its normal physiological function (Higgins et al., 2005). However, the development of FMT today is not only as a therapeutic method, but also has more applications, such as adjusting the intestinal flora of humans or animals through FMT to improve the growth rate or production performance, or altering the metabolism of obese populations as well as the establishment of disease models, and so on (Elangovan et al., 2022). Remarkably, this method traces its origins back 1,700 years to the fourth century when a Chinese scientist named Ge Hong administered oral suspensions of human feces to treat patients with food poisoning and severe diarrhea (McDonald et al., 2005). The earliest documented case of FMT being employed to treat *C. difficile* infection (CDI) dates back to 1983 (Higgins et al., 2005). FMT has proven therapeutic applications in a range of gastrointestinal disorders, including CDI, chronic fatigue syndrome, Inflammatory Bowel Disease (IBD), Irritable Bowel Syndrome (IBS), and Ulcerative Colitis (UC) (Lewin, 2001; Cammarota et al., 2014; Rossen et al., 2015; Rodrigues et al., 2017). Moreover, research by Lin et al. (2018) has revealed that FMT can enhance metabolic processes in the gut. In the domain of aquaculture production, FMT has also demonstrated potential by improving the intestinal flora of livestock and poultry, consequently impacting their growth performance. A study conducted by Rosa et al. (2021) involved the transplantation of feces from adult dairy cows into newborn calves. The result showed that calves in the FMT group exhibited a gradual increase in body weight from birth to weaning, surpassing those in the CK group. This improvement was attributed to the alteration in the relative abundance of the *Firmicutes* and *Bacteroidetes*, suggesting that the interaction of transplanted intestinal flora played a role in enhancing the growth performance of the recipient calves. Similarly, Wang et al. (2019) conducted research by transplanting feces from healthy adult pigs into weaned piglets, with results that FMT significantly improved the growth performance of the pigs. The intestinal microbial composition and stage characteristics of the recipients did not change significantly, but the abundance of *Streptococcus* spp. and *Clostridium* spp. increased, which was hypothesized to be likely to promote the growth of the animals. The use of donor chickens with higher feed utilization for FMT to establish a more desirable microbiota in the intestinal tract of recipient chickens may serve as a new way to regulate microbial colonization in chickens at early stage (Siegerstetter et al., 2018).

Previous research has demonstrated that C57BL/6 mice tend to exhibit more favorable body weight characteristics when compared to DBA/2 mice (Xi et al., 2011). To investigate whether this variance might be linked to differences in their intestinal microbiota, we embarked on a series of experiments. For the first group (T1 group), we transplanted fecal bacteria from C57BL/6 mice into DBA mice. This was done to assess whether alterations in the intestinal flora could impact body weight in DBA mice. Building upon these findings, in the second group (T2 group), we transplanted fecal bacteria from high-fat diet-fed C57BL/6 mice of the same age into the intestines of DBA mice. Our goal was to explore how the high-fat diet affected the intestinal microbiota of C57BL/6 mice and the subsequent consequences on DBA mice’s body weight. Furthermore, to investigate whether factors beyond genetic predisposition contribute to the differential growth rates observed in rats concerning their gut microbiota, we introduced an additional experimental group. In this third group (T3 group), we transplanted fecal bacteria from Wistar rats into the intestines of DBA mice. This was done to assess the potential role of gut microorganisms in the growth disparities between these species. During the test period, donors in the T1 and T3 groups were provided with a normal diet, and donors in the T2 group were provided with a high-fat diet.

## 2 Materials and methods

All procedures of animal experiments were carried out based on protocols approved by the Animal Care Advisory Committee of Zhejiang University, Hangzhou, China (No. ZJU20220438).

### 2.1 Animals

Use 10 SPF male adult C57BL/6 mice at 6 weeks old, 10 SPF male adult high-fat diet-fed C57BL/6 mice at 6 weeks old, and 6 SPF male adult Wistar rats at 10 weeks old (Hans Biotechnology Co., Ltd., Hangzhou, China) as donors, male DBA mice (bred in SPF-grade conditions at Hans Biotechnology Co., Ltd., Hangzhou, China) at 7 days old as accepters. All DBA mice were housed with their mothers before weaning, and after weaning, they were housed in separate cages, and after 1 week of acclimatization feeding, mice of similar body weight were selected for the experiment. All animals were housed under controlled conditions, including a constant temperature range of 21–24°C, humidity maintained at 50–60%, and exposure to natural lighting. Water was provided *ad libitum* to the animals throughout the study. Donors in the T1 and T3 groups were provided with a normal diet, and donors in the T2 group were provided with a high-fat diet.

All experimental procedures were conducted in strict adherence to the guidelines for animal care and use. The research protocols were approved by the Committee for Animal Research at Zhejiang University, Hangzhou, China (approval number ZJU20220438).

### 2.2 Experimental protocol

After a one-week acclimation period, the DBA mice were randomly divided into four groups, each consisting of 12 mice: Control group (CK); C57BL/6 mice fecal microbiota transplantation (FMT) group (T1); High-fat C57BL/6 FMT group (T2); Wistar rats FMT group (T3). Fresh fecal samples were collected daily from C57BL/6 mice, high-fat C57BL/6 mice, and Wistar rats to serve as donors. The collected fecal material was placed in sterile tubes and homogenized using sterile normal saline (Sinopharm Chemical Reagent Co. Ltd., China). This mixture was then subjected to centrifugation at 6,000 × g for 10 min, followed by filtration through a 70-μm filter (Nest, China). The resultant material was quantified to achieve an optical density (OD) at 600 nm of 1.0, corresponding to an estimated bacterial concentration of 10^9^ colony-forming units (CFU) per milliliter. Subsequently, the fecal bacterial slurry was diluted to a final concentration of 5.0 × 10^9^ CFU/mL using a 30% sterile medical glycerin phosphate-buffered saline (PBS) (Sinopharm Chemical Reagent Co. Ltd., China). Any remaining fecal fluid was stored at −80°C for future use. The mice in the CK group were administered sterile normal saline via gavage once a day for a duration of 28 days using disposable sterile syringes (1 mL, DKBT, China) with sterile gavage needles (45 mm, Shenzhen Longreen Lighting Co., Ltd., China) (Zhang et al., 2020; Elangovan et al., 2022). In contrast, the mice in the T1 group received fecal fluid from normal C57BL/6 mice, the T2 group received fecal fluid from high-fat C57BL/6 mice, and the T3 group received fecal fluid from Wistar rats through gavage over the same 28-day period.

### 2.3 Sample collection

At the end of the experiment, the finishing mice were fasted for 24 h (6-h daytime and 16-h overnight) (Zhang et al., 2016) and at which time, blood samples were collected at baseline into a vacuum tube. Mice were anesthetized by intraperitoneal injection of 80 mg/kg sodium pentobarbital and were euthanized by intraperitoneal injection of 200 mg/kg sodium pentobarbital under deep anesthesia. The colon and liver tissues were collected immediately from all mice. After euthanasia of the mice, the same intestinal segments from the posterior half of the colon were taken and their contents squeezed into cryopreservation tubes, which were immediately stored at −80°C for subsequent sequencing. A part of the liver and colon was fixed using 4% paraformaldehyde solution, the rest of liver and colon tissue was saved at −80°C.

### 2.4 Growth performance indicators

The mice and food intakes were meticulously weighed at the end of each week, specifically on the 1st, 2nd, 3rd, 4th, and 5th weeks. This data was then used to calculate both the daily weight gain and the daily feed intake of the mice.

### 2.5 Serum biochemical indicators

The level of serum biochemical indicators were measured by Albumin (ALB) assay kit, Total Cholesterol (TC) assay kit, Triglyceride (TG) assay kit and Superoxide Dismutase (SOD) assay kit (Nanjing Jiancheng Institute of Bioengineering, China), as well as the inflammatory markers of the mice including Immunoglobulin A (IgA), Interleukin-1β (IL-1β), Interleukin-6 (IL-6), Interleukin-10 (IL-10), Interleukin-22 (IL-22), Tumor Necrosis Factor-α (TNF-α), Chemokine (C-X-C motif) ligand 1 (CXCL1) and Chemokine (C-X-C motif) ligand 2 (CXCL2) (Jiangsu Meimian Industrial Co., Ltd., China) were measured with assay kits according to the manufacturer’s instructions with UV–VIS Spectrophotometer (UV1100, MAPADA, Shanghai, China) following the manufacturer’s instructions.

### 2.6 Microbial 16S rRNA gene sequencing library construction and sequencing

#### 2.6.1 DNA extraction and PCR amplification

Total microbial genomic DNA was extracted from colon contents samples using the E.Z.N.A.^®^ soil DNA Kit (Omega Bio-tek, Norcross, GA, United States) following the manufacturer’s instructions. The quality and concentration of DNA was determined by 1.0% agarose gel electrophoresis and a NanoDrop^®^ ND-2000 spectrophotometer (Thermo Scientific Inc., United States) and stored at −80°C before further use. The V3-V4 hypervariable region of the bacterial 16S rRNA gene was amplified using primer pairs 341F (5′-CCTAYGGGRBGCASCAG-3′) and 806R (5′-GGACTACHVGGGTWTCTAAT-3′) on an ABI GeneAmp^®^ 9700 PCR thermocycler (ABI, CA, United States) (Liu et al., 2016). The PCR reaction mixture contained 4 μL 5 × Fast Pfu buffer, 2 μL 2.5 mM dNTPs, 0.8 μL each primer (5 μM), 0.4 μL Fast Pfu polymerase, 10 ng template DNA and ddH2O to a final volume of 20 μL. PCR amplification cycling conditions were as follows: initial denaturation at 95°C for 3 min, followed by 27 cycles of denaturation at 95°C for 30 s, annealing at 55°C for 30 s and extension at 72°C for 45 s, and a single extension at 72°C for 10 min, terminating at 4°C. All samples were amplified in triplicate. The PCR product was extracted from a 2% agarose gel and purified using the AxyPrep DNA Gel Extraction Kit (Axygen Biosciences, Union City, CA, United States) according to the manufacturer’s instructions and quantified using the Quantus^™^ Fluorometer (Promega, United States).

#### 2.6.2 Illumina MiSeq sequencing

The main stages of library construction using the NEXTFLEX Rapid DNA-Seq Kit (Bioo Scientific, United States), including linkage of junctions, removal of junction self-associated fragments, PCR amplification for library template enrichment, and PCR product recovery. The sequencing process was performed using Illumina’s Miseq PE300 (Illumina, United States), which involves complementing one end of the DNA fragment with the junction bases embedded in the chip to form a fixed bridge structure, followed by PCR amplification to produce DNA clusters, which are subsequently linearized into single strands. Sequence information of the template DNA fragments is obtained by scanning the surface of the reaction plate with a laser, synthesizing only one base per round, and by chemical cleavage and counting fluorescent signals (Majorbio Bio-Pharm Technology Co. Ltd., Shanghai, China^1^).

#### 2.6.3 Amplicon sequence processing and analysis

Following the demultiplexing process, the sequences produced underwent quality filtration with fastp (version 0.19.6) (Chen et al., 2018) and were merged with FLASH (version 1.2.11) (Magoč and Salzberg, 2011). Next, using DADA2 (Callahan et al., 2016) plugin within the Qiime2 (Bolyen et al., 2019) (version 2020.2) pipeline with recommended parameters, the high-quality sequences were de-noised. This process obtained single nucleotide resolution based on error profiles within samples, resulting in the creation of amplicon sequence variants (ASVs) using the DADA2 denoised sequences. Taxonomic assignments of ASVs were made using the Naive Bayes (or Vsearch or Blast) consensus taxonomy classifier implemented in Qiime2 along with the SILVA 16S rRNA database (v138).

### 2.7 Transcriptomics

Colon RNA samples from 20 different male mice were used for mRNA sequencing.

#### 2.7.1 Extraction of total RNA

Total RNA was extracted from tissue samples, and the concentration and purity of the extracted RNA were examined using Nanodrop2000, RNA integrity was detected by agarose gel electrophoresis, and RNA integrity number (RIN) value was determined by Agilent2100. Total RNA amount ≥1ug, concentration ≥35 ng/μL, OD260/280 ≥ 1.8, OD260/230 ≥ 1.0 were required for single library construction.

#### 2.7.2 Oligo dT mRNA enrichment

The 3′ end of eukaryotic mRNA has the structure of ployA tail, and the magnetic beads with Oligo (dT) are utilized to perform A-T base pairing with ployA. Using magnetic beads with Oligo (dT) to perform A-T base pairing with ployA, mRNA can be isolated from total RNA and used to analyze transcriptome information.

#### 2.7.3 Fragmented mRNAs

The Illumina platform sequences short fragments, and the mRNAs obtained from enrichment are complete RNA sequences, averaging several kb in length, and therefore need to be randomly interrupted. By adding fragmentation buffer, mRNA can be randomly fragmented, and small fragments of about 300 bp can be separated by magnetic bead screening.

#### 2.7.4 Reverse cDNA synthesis

Under the action of reverse transcriptase, six-base random hexamers are added, and mRNA is used as a template for reverse transcription. One-stranded cDNA is synthesized by adding six-base random hexamers under the action of reverse transcriptase, using mRNA as a template for reverse transcription, followed by two-stranded synthesis to form a stable double-stranded structure.

#### 2.7.5 Linkage adaptor

The double-stranded cDNA structure has sticky ends, which are made flat by adding End Repair Mix, followed by the addition of an “A” base at the 3′ end. Then an “A” base was added to the 3′ end to connect the Y-shaped connector.

Finally, we performed up-sequencing on the illumination platform (Majorbiobio, Shanghai, China) (Schoeler and Caesar, 2019). Library enrichment, PCR amplification of 15 cycles; (Yu et al., 2019) 2% agarose gel recovery of the target bands; (Elangovan et al., 2022) TBS380 (Picogreen) quantification, mixing the data proportionally on the machine; (McDonald et al., 2005) Bridge PCR amplification on cBot to generate clusters; (Higgins et al., 2005) Sequencing on Illumina platform (PE library, read length 2 × 150 bp) (MiSeq Reagent Kit v3, Illumina, United States).

### 2.8 Statistical analysis

All data except for the 16S RNA results were analyzed by one-way analysis of variance (ANOVA) using SPSS statistical software (Ver. 20.0 for windows, SPSS, Inc., Chicago, IL, United States). Differences among treatments were examined using the Tukey–Kramer’s multiple range tests, which were considered significant when the *p*-value was less than 0.05. Results are presented as means alongside their pooled standard errors of means (SEM).

For 16S RNA analysis, based on the ASVs information, α diversity indices (including the Sobs index, Chao richness estimator, Shannon diversity index, and Simpson index) between the mice of three groups were calculated to evaluate microbial species richness and evenness (Schloss et al., 2009). The similarity among the microbial communities in different samples was determined by principal coordinate analysis (PCoA) based on Bray–curtis dissimilarity using Vegan v2.5-3 package. Use Wilcox rank-sum test analysis to analyze which bacteria differ in the intestinal flora of different groups of mice at the phylum level and genus level, respectively. Using the two-sample *t*-test, we analyzed the community abundance data and rigorously determined the significance of differences in species abundance between the two groups of sampled microbial communities. The hypothesis of species between the groups was tested using R-3.3.1 (stat) software and enabled identification of the species that exhibited significant differences between the groups (Mojorbio, Shanghai, China). The raw sequencing reads were deposited into the NCBI Sequence Read Archive (SRA) database (Accession Number: PRJNA1050037).

## 3 Results

### 3.1 Impact of FMT on the growth performance of DBA mice

**Figure 1:**
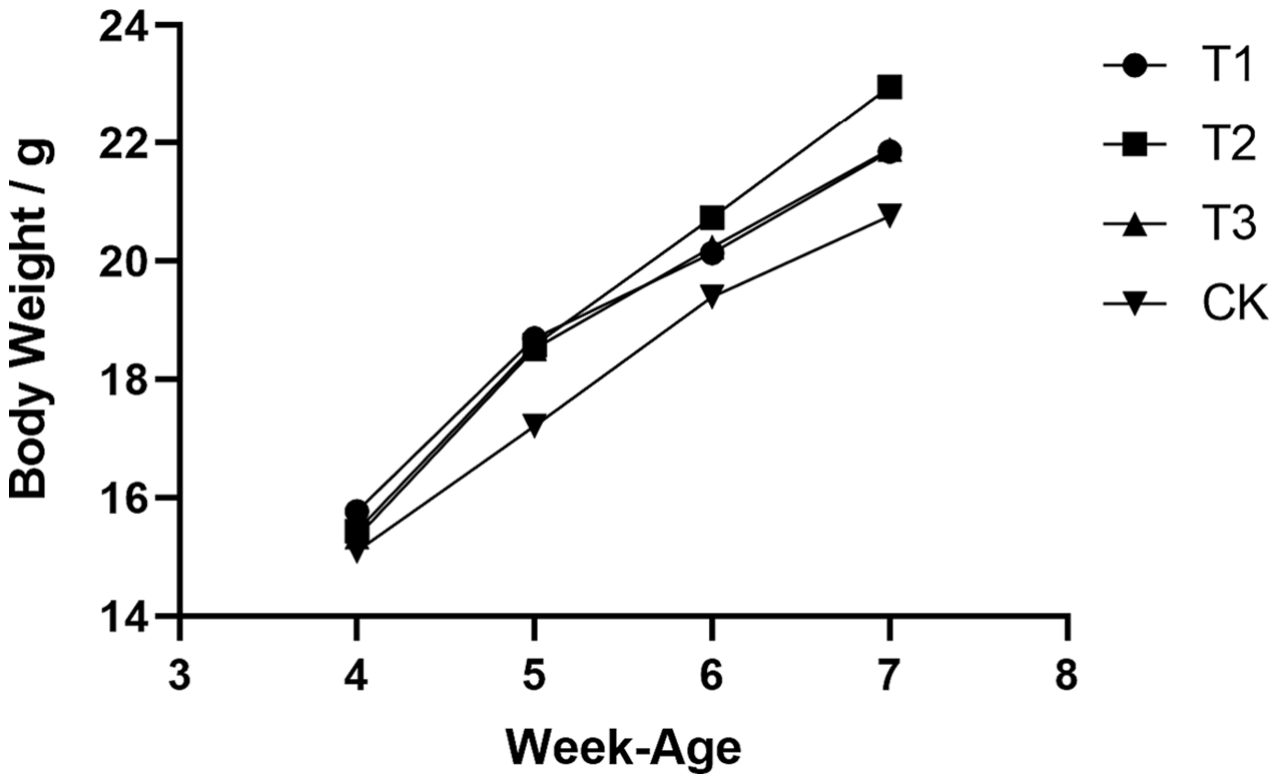
Comparison of growth curves of four groups of mice. CK: DBA mice accepting saline gavage; T1: DBA mice receiving FMT from C57BL/6 mice; T2: DBA mice receiving FMT from high-fat C57BL/6 mice; T3: DBA mice receiving FMT from Wistar rats.

Initial body weights of DBA mice were measured at 4 weeks of age, following 28 days of FMT gavage, the final body weight (FBW) and average daily gain (ADG) of mice that received FMT from C57BL/6 mice, high-fat C57BL/6 mice, and Wistar rats exhibited substantial increased compared to the CK group, and the overall growth curve was better than that of the mice in CK group (Figure 1). These results suggested that FMT indeed enhances the growth performance of DBA mice. Moreover, it is worth noting that the growth performance improvement observed in DBA mice that received FMT from high-fat C57BL/6 mice was significantly greater than that in DBA mice receiving FMT from normal C57BL/6 mice and Wistar rats. This observation implied that the high-fat model also induces notable alterations in the intestinal flora of mice.

### 3.2 Impact of FMT on immune function in DBA mice

**Figure 2:**
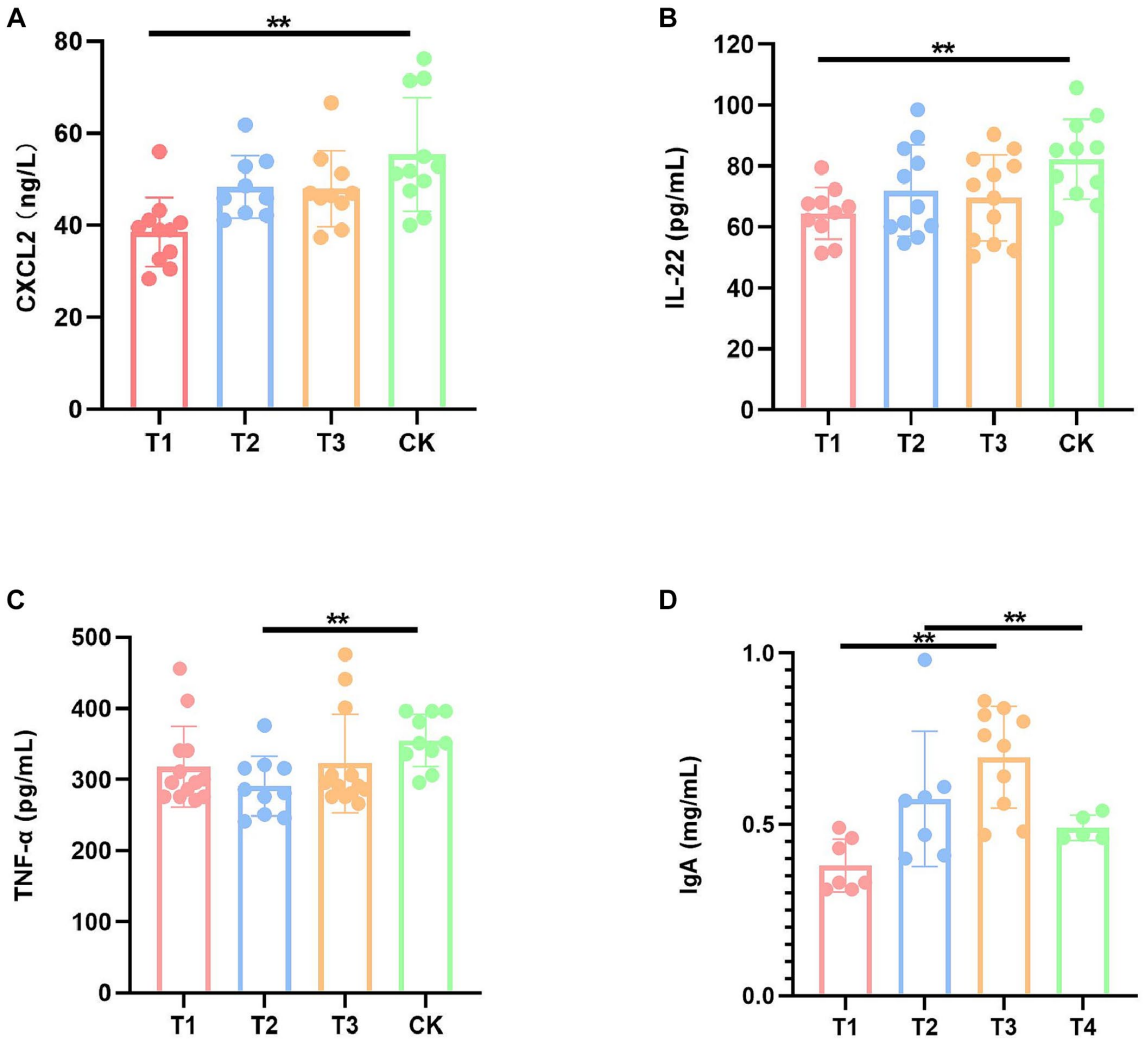
Immunization factors with significant differences of mice in four groups. **(A)** Serum CXCL2 levels. **(B)** Serum IL-22 levels. **(C)** Serum TNF-α levels. **(D)** Serum IgA level. CK: DBA mice accepting saline gavage; T1: DBA mice receiving FMT from C57BL/6 mice; T2: DBA mice receiving FMT from high-fat C57BL/6 mice; T3: DBA mice receiving FMT from Wistar rats. **P < 0.01.

In order to evaluate the effect of FMT on the immune function of DBA mice, we performed a series of serum ELISA assays, including CXCL1, CXCL2, TNF-α, IL-1β, IL-6, IL-22, IL-10, and IgA (Supplementary Table S2). The results demonstrated a significant reduction in the levels of CXCL2 and IL-22 in the T1 group compared to the CK group, a significant decrease in TNF-α levels in the T2 group compared to the CK group, a significant decrease in the levels of IL-22 in the T3 group compared to the CK group, and a significant increase in IgA levels (Figure 2).

### 3.3 Impact of FMT on lipid profiles, liver function, and antioxidant capacity in DBA mice

**Figure 3:**
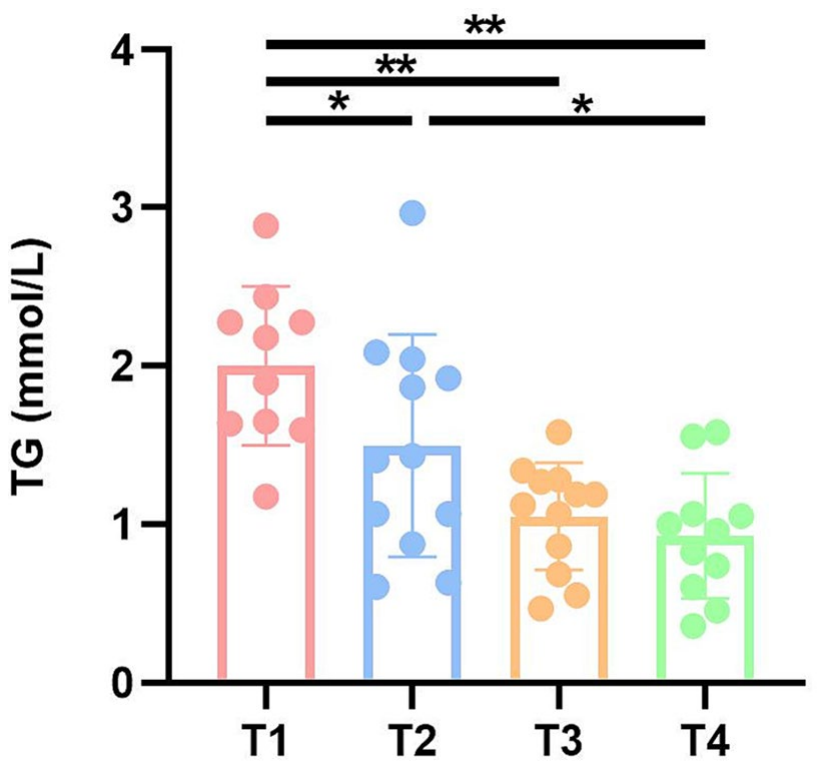
TG levels of mice in four groups. CK: DBA mice accepting saline gavage; T1: DBA mice receiving FMT from C57BL/6 mice; T2: DBA mice receiving FMT from high-fat C57BL/6 mice; T3: DBA mice receiving FMT from Wistar rats. **P < 0.05, **P < 0.01.

To investigate the effects of fecal microbiota transplantation on lipids, liver function and antioxidant capacity in DBA mice, serum TG, TC, TP, ALB, and SOD were measured in four groups of mice (Supplementary Table S3). Notably, serum triglyceride (TG) levels exhibited a significant increase in both the T1 and T2 groups compared to the CK group (Figure 3).

### 3.4 Microbial 16S rRNA gene sequencing library construction and sequencing

**Figure 4:**
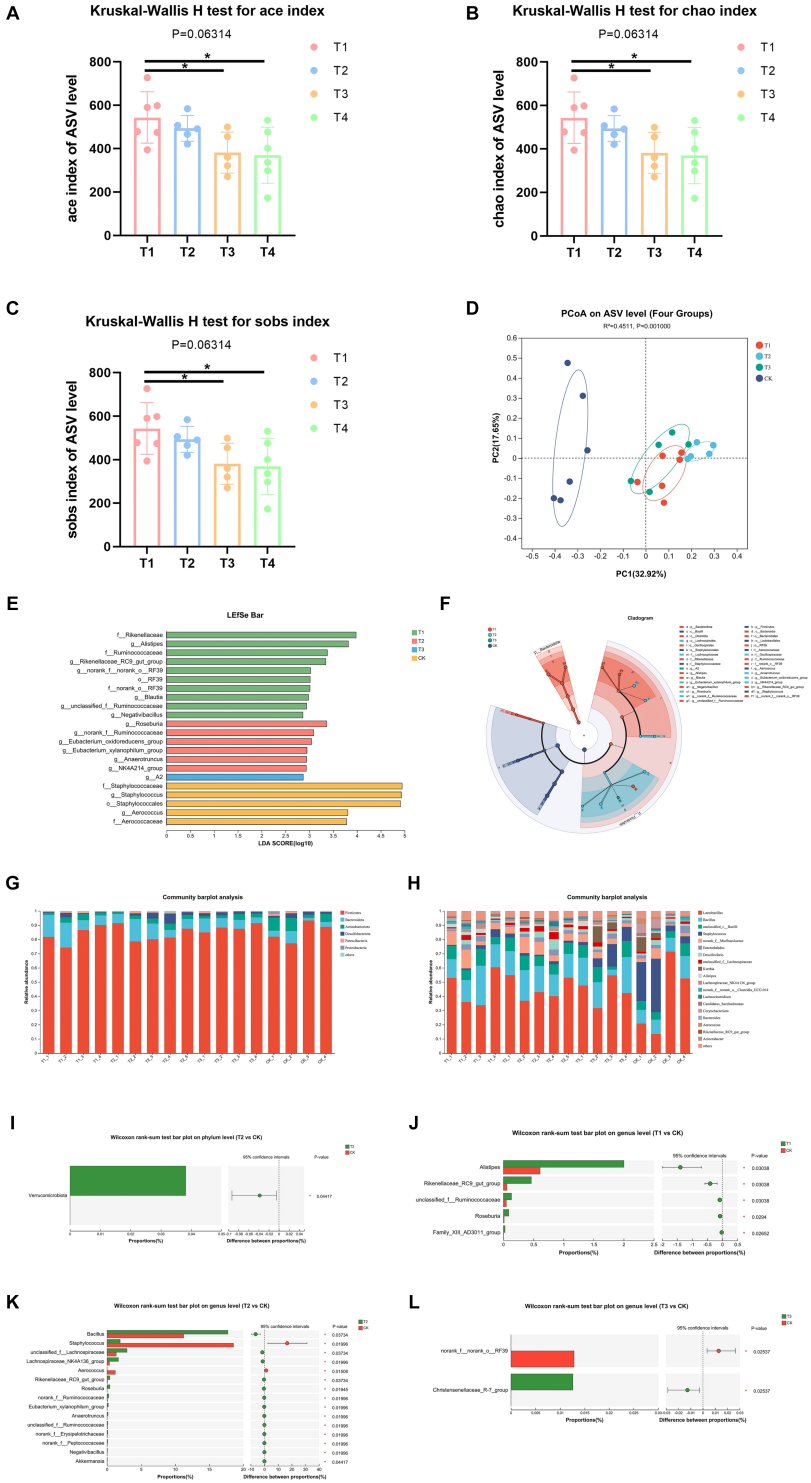
FMT modulated microbiota composition. **(A–C)** Ace, chao, and sobs index of ASV level. **(D)** β-diversity between experiment groups and CK group on ASV level. **(E,F)** LEfSe analysis of four groups. o: Order, f: Family, g: Genus. **(G,H)** The relative abundance of bacteria on phylum and genus. **(I)** Comparative analysis of the relative abundance of bacteria at phylum level between T2 group and CK group. **(J–L)** Comparative analysis of the relative abundance of bacteria at genus level between experimental groups and CK group; CK: DBA mice accepting saline gavage; T1: DBA mice receiving FMT from C57BL/6 mice; T2: DBA mice receiving FMT from high-fat C57BL/6 mice; T3: DBA mice receiving FMT from Wistar rats.

Information on the richness, diversity and coverage of species in the community was obtained through α diversity index analysis, and the test of difference between groups was applied to detect whether the values of Α diversity index were significantly different between every two groups and above. The results showed a significant increase in α-diversity indices, including ace, chao and sobs indices of the gut microbiota of T1 group than T3 and CK group (Figures 4A–C), which suggested that FMT from normal C57BL/6 mice increased bacterial richness of DBA mice (as determined by rising ace, chao and sobs indices). Then, we analyzed the β-diversity with the Bray-Curtis principal coordinate analysis (PCoA) on ASV level, to identify potential principal components that influence differences in the composition of the sample communities through dimensionality reduction (Figure 4D). PCoA exposed significant difference in gut microbiota between T1 group and CK group (*R* = 0.622, *p* = 0.003). Also, T2 group and CK group showed significant difference in gut microbiota (*R* = 0.744, *p* = 0.004), same was between T3 group and CK group (*R* = 0.507, *p* = 0.004). These results indicated that FMT modulates gut microbiota composition of DBA mice.

In order to identify high-dimensional biomarkers and reveal genomic features, we performed Linear discriminant analysis Effect Size (LFfSe) analysis of the flora in the mouse gut from the phylum level to the genus level. Figures 4E–F shows the results for all LDA values greater than 2. After FMT, the four groups of mice with important roles in the gut of microbial taxa differed significantly. Compared with the control group, mice in group T1 were significantly enriched in RF39 at the Order level (LDA = 3.014, *p* = 0.022), at the Family level Ruminococcaceae (LDA = 3.375, *p* = 0.047), Rikenellaceae (LDA = 3.980, *p* = 0.21) and norank_o_RF39 (LDA = 3.014, *p* = 0.022) were significantly enriched, at the Genus level *unclassified_f_Ruminococcaceae* (LDA = 2.943, *p* = 0.011), *Negativibacillus* (LDA = 2.865, *p* = 0.020), Blautia (LDA = 2.981, *p* = 0.036), *Alistipes* (LDA = 3.817, *p* = 0.047), *g_norank_f_norank_o_RF39* (LDA = 3.025, *p* = 0.022) and *g_Rikenellaceae_RC9_gut_group* (LDA = 3.345, *p* = 0.024) were significantly enriched. T2 group only enriched at Genus level, which were *Roseburia* (LDA = 3.364, *p* = 0.009), *norank_f_Ruminococcaceae* (LDA = 3.094, *p* = 0.026), *Eubacterium_xylanophilum_group* (LDA = 2.950, *p* = 0.017), *Eubacterium_oxidoreducens_group* (LDA = 3.045, *p* = 0.027), *NK4A214_group* (LDA = 2.936, *p* = 0.049) and *Anaerotruncus* (LDA = 2.942, *p* = 0.033). T3 group only enriched in Genus *A2* (LDA = 2.871, *p* = 0.044).

Thereafter, we assessed the relative abundance of bacteria on phylum and genus levels. Firmicutes and Bacteroidota were the most abundant, accounting for over 80% of all microorganisms in all groups at Phylum level (Figure 4G). At genus level, Lactobacillus and Bacillus were the most abundant, accounting for over 50% of all microorganisms in T1, T2, and CK groups, meanwhile Lactobacillus and Staphylococcus were the most abundant accounting for over 50% of all microorganisms in T3 group (Figure 4H).

In order to assess the level of significance of the differences in species abundance and to obtain information on the species that were significantly different between the two groups, we utilized the between-groups test of variance methodology, applying rigorous statistical methods based on the community abundance data obtained to test hypotheses about the species between the two groups of sampled microbial communities. The results showed that only T2 group and CK group showed significant difference at Phylum level, which was a significant rise in Verrucomicrobiota (*p* = 0.045, Figure 4I). At genus level, the abundance values of *Alistipes*, *Rikenellaceae_RC9_gut_group*, *unclassified_f_Ruminococcaceae*, *Roseburia*, and *Family_XIII_AD3011_group* in group T1 were all significantly increased relative to CK group (Figure 4J). The abundance of *Bacillus*, *unclassified_f_Lachnospiraceae*, *Lachnospiraceae_NK4A136_group*, *Rikenellaceae_RC9_gut_group*, *Roseburia*, *norank_f_Ruminococcaceae*, *Eubacterium_xylanophilum_group*, *Anaerotruncus*, *unclassified_f_Ruminococcaceae*, *norank_f_Erysipelotrichaceae*, *norank_f_Peptococcaceae*, *Negativibacillus* and *Akkermansia* of T2 group all showed a significant increase in abundance values relative to the CK group, while *Aerococcus* and *Staphylococcus* showed a decrease in abundance values relative to the CK group (Figure 4K). As is shown in Figure 4L, T3 group significantly decreased the abundance of *norank_f_norank_o_RF39* and increased the abundance of *Christensenellaceae_R-7_group*. Thus, FMT altered the gut flora composition of DBA mice.

### 3.5 Transcriptome construction and sequencing

**Figure 5:**
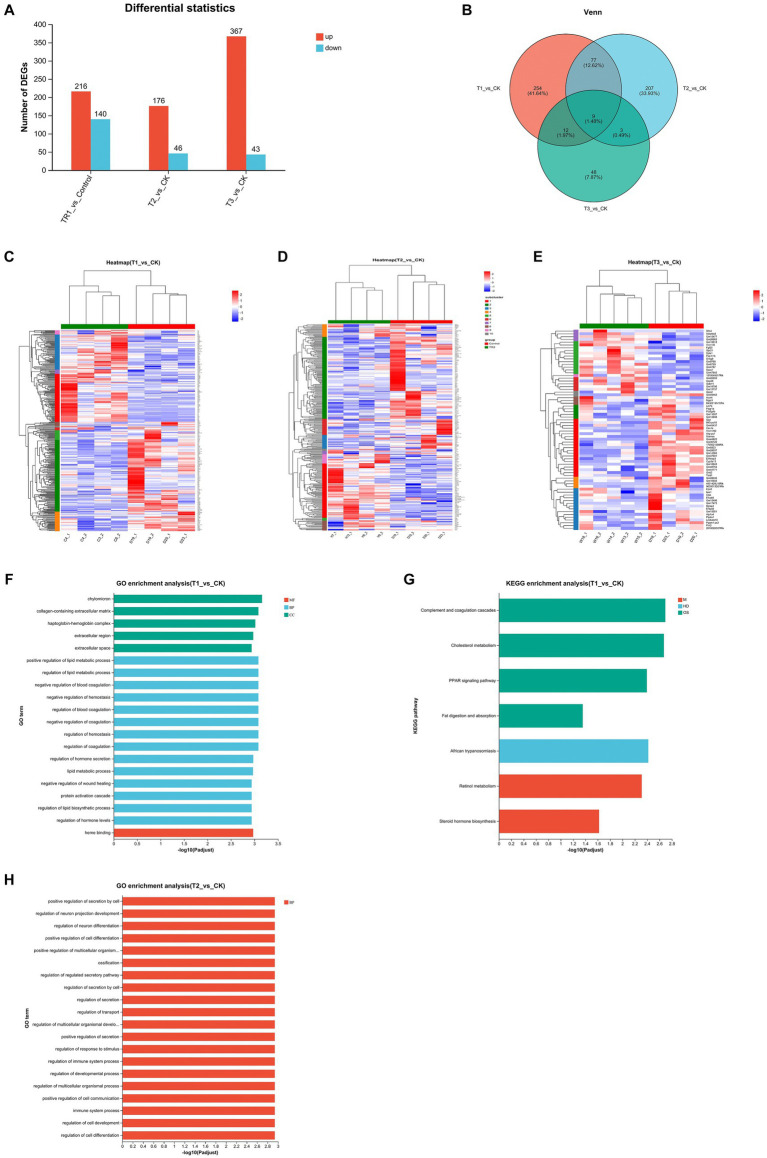
Analysis of DEGs in the colon among four groups. **(A,B)** Differential gene expression statistics. **(C–E)** Heatmap showing the differential expression of genes among the groups. **(F–H)** GO and KEGG pathway enrichment analysis of DEGs; CK: DBA mice accepting saline gavage; T1: DBA mice receiving FMT from C57BL/6 mice; T2: DBA mice receiving FMT from high-fat C57BL/6 mice; T3: DBA mice receiving FMT from Wistar rats.

Since this experiment involved FMT across strains and breeds, in order to investigate whether changes in intestinal flora would alter gene expression in the gut, we took colon tissues from four groups of mice for transcriptome sequencing. By quantitatively analyzing the genes sequenced in the four groups of mice and obtaining their reads, the samples were analyzed for differential expression of genes between groups, identifying differentially expressed genes between groups, and then studying the functions of the differential genes (Figures 5A,B). Mice in group T1 had a total of 356 differential genes compared to CK group, with 216 up-regulated and 140 down-regulated genes. Mice in group T2 had a total of 222 differential genes compared to CK group, with 176 up-regulated and 46 down-regulated genes. Mice in group T3 had a total of 416 differential genes compared to CK group, with 367 up-regulated and 43 down-regulated genes.

Based on the information about the expression number of genes in different samples, the distance between genes and samples is calculated, and then an iterative method was used to classify the genes and samples, and the differentially expressed genes between each experimental group and the CK group were plotted as a heatmaps (Figures 5C–E). Acceptance of FMT from normal C57BL/6 mice up-regulated cholesterol metabolism related genes (Apoh, Apob, Apoc3, Apoa1, Apoa2), PPAR signaling pathway related genes (Apoa2, Apoa5, Apoc3, Apoa1, Fabp1) and complement and coagulation cascades related genes (Fga, C8b, Fgb, Fgg, C8a, Plg) in DBA mice. Acceptance of FMT from high-fat C57BL/6 mice similarly upregulated genes that increase the cancer related genes (Mycn, C4bp) (Figure 5D). The Pla2g3b gene was down-regulated by fecal microbiota transplants from Wistar rats (Figure 5E).

GO functional and KEGG pathway enrichment analysis were performed with the DEGs among the four groups. Enrichment results of top20 were shown at padjust <0.05, where T1 group versus CK group had enrichment results in both GO and KEGG pathway, T2 group versus CK group had only GO enrichment results, and T3 group versus CK group did not produce enrichment results in both GO and KEGG pathway. The results showed that the DEGs between T1 group and CK group were categorized into three groups [molecular function (MF), biological process (BP), and cellular component (CC)] based on GO enrichment analysis, and many of them were associated with lipid metabolic process, such as positive regulation of lipid metabolic process, regulation of lipid metabolic process, lipid metabolic process, regulation of lipid biosynthetic process, regulation of lipid biosynthetic process in BP. There were also many related to hemostasis and coagulation, such as negative regulation of blood coagulation, negative regulation of hemostasis, regulation of blood coagulation, negative regulation of coagulation, regulation of hemostasis, regulation of coagulation in BP, and heme binding in CC. Other high enriched GOs were chylomicron, collagen-containing extracellular matrix, haptoglobin-hemoglobin complex, extracellular region and extracellular space in MF, regulation of hormone secretion, negative regulation of wound healing, protein activation cascade and regulation of hormone levels in BP (Figure 5F). KEGG pathway enrichment showed that the DEGs between T1 group and CK group were categorized into three groups [metabolism (M), human disease (HD), organism system (OS)]. And many of them were associated to fat and sterol such as Cholesterol metabolism, Fat digestion and absorption in OS, and Steroid hormone biosynthesis of HD. Other high enriched KEGG pathways were African trypanosomiasis of M and Complement and coagulation cascades, PPAR signaling pathway in OS, together with Retinol metabolism in HD (Figure 5G). The DEGs between T2 group and CK group were categorized into biological process (BP), and most of them are related to the regulation of cellular activity (positive regulation of secretion by cell, positive regulation of cell differentiation, positive regulation of multicellular organismal process, regulation of secretion by cell, regulation of multicellular organismal development, regulation of multicellular organismal process, positive regulation of cell communication, regulation of cell development, regulation of cell differentiation). Others were related to neuron-related regulation, secretory regulation and the immune system (Figure 5H).

### 3.6 Differentially expressed genes related to the composition of gut bacterial communities

**Figure 6:**
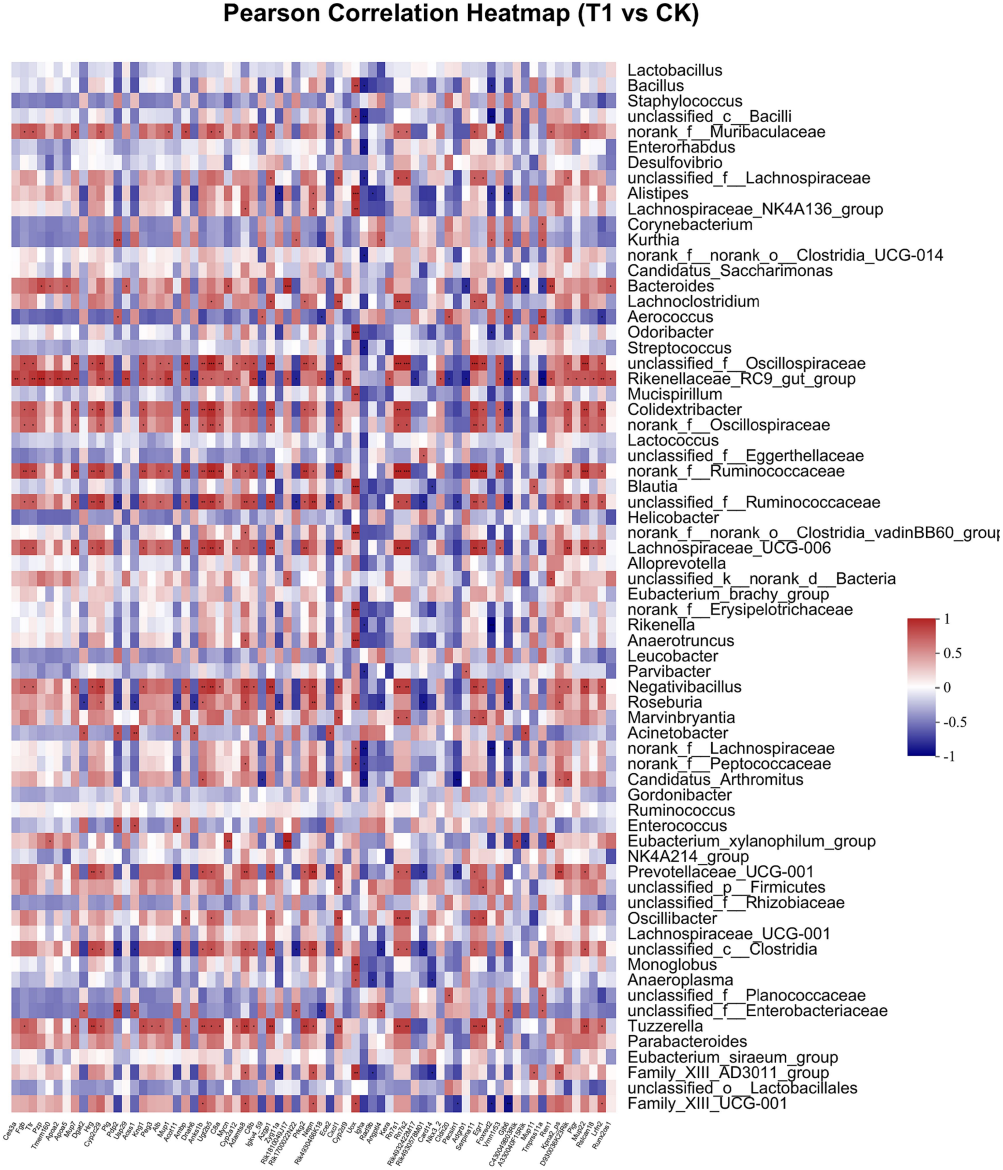
Pearson Correlation Heatmap of microbial communities on the genus level and DEGs between T1 and CK groups.

**Figure 7:**
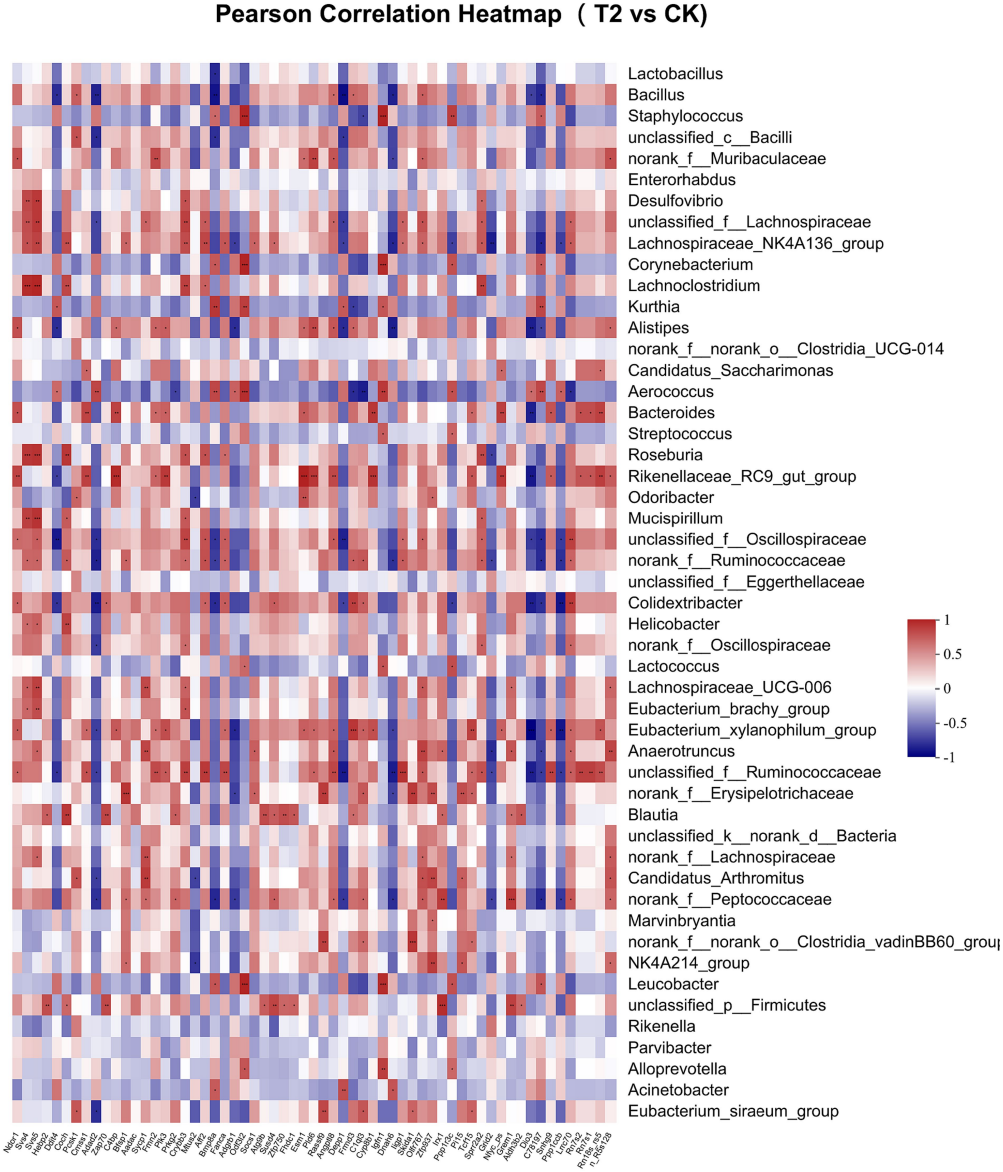
Pearson Correlation Heatmap of microbial communities on the genus level and DEGs between T2 and CK groups.

**Figure 8:**
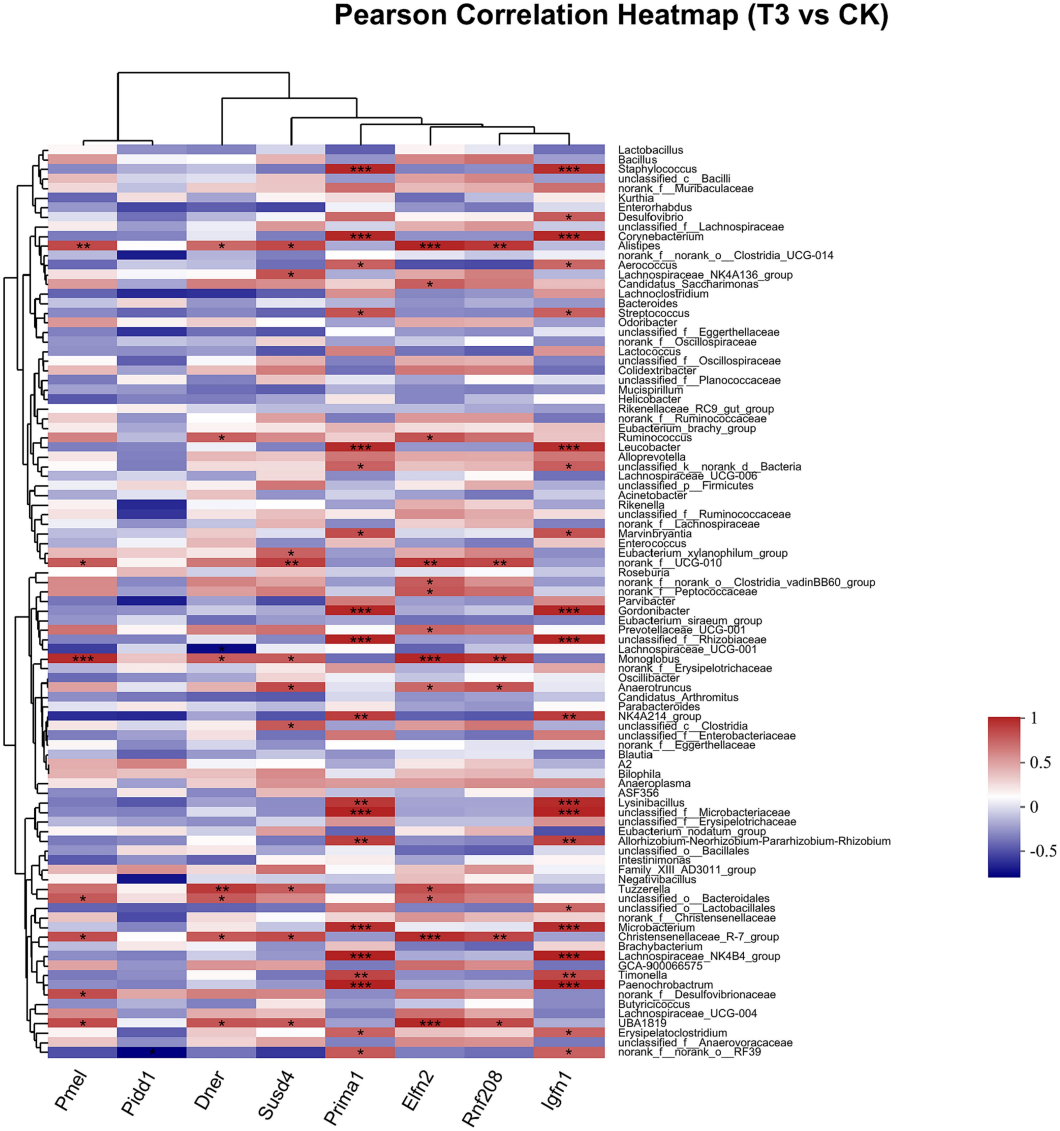
Pearson Correlation Heatmap of microbial communities on the genus level and DEGs between T3 and CK groups.

Pearson correlation was used to infer the relationship between the composition of the microbial communities on the genus level and DEGs. The top candidate genes with genus-level relationships with the bacterial community are listed in Supplementary Tables S4, S5. Correlation analysis of the 357 differential genes in the T1 and CK groups with the five genus-level differential flora yielded 111 genes with significant correlations, most of which had KEGG functional annotations related to fatty acid metabolism (Apoa5, Angptl4), fat digestion and absorption (Dgat2), cholesterol metabolism (Apoa2, Kng1), inflammation regulation (Cyp2c29) and coagulation (C8b, Fgb, C8a, Plg) (Figure 6). Correlation analysis of 221 differential genes from the T2 and CK groups with 15 genus-level differential flora yielded 111 genes with significant correlations, most of which had KEGG functional annotations related to fatty acid metabolism (Cyp8b1), cholesterol metabolism (Angptl8), and coagulation (C4bp) (Figure 7). Correlation analysis of 416 differential genes from the T3 and CK groups with 2 genus-level differential flora yielded 7 genes with significant correlations, most of which had KEGG functional annotations related to cancer and cell apoptosis (Prima1, Pidd1, Pmel, Dner, Rnf208, Igfn1) (Sun et al., 2009; Izetti et al., 2014; Weiler et al., 2022) and Nervous-System Function (Susd4, Elfn2) (Figure 8).

## 4 Discussion

### 4.1 Relationship between the gut bacterial community structure and growth performance

Normal steady-state intestinal flora is closely related to host health and forms a reciprocal relationship with the host through complex interactions. Its unique community structure and metabolites are essential for the regulation of host metabolism, growth and development, pathogen resistance, immunomodulation, adaptation, and evolution (Bäckhed et al., 2005; Engel et al., 2012; Ezenwa et al., 2012; Zhang et al., 2016). To investigate whether the effect of fecal transplantation on the growth performance of DBA mice is related to the intestinal flora, mice from three experimental groups and CK group were cultured in similar environments during the reproductive period to explore the differences in the structure of their intestinal bacterial communities. The PCoA analysis of the bacterial flora revealed that the three experimental groups and CK group were clearly distinguishable. The results showed that the T1 group significantly enhanced five dominant bacteria genera including the genera *Alistipes*, *Rikenellaceae_RC9_gut_group*, *unclassified_f_Ruminococcaceae*, *Roseburia*, and *Family_XIII_AD3011_group*. *Alistipes* and *Rikenellaceae_RC9_gut_group* belong to Bacteroidetes. It has been shown that *Alistipes* may be protective against certain diseases, including liver fibrosis, cancer immunotherapy and cardiovascular disease. In addition to this, *Alistipes* spp. have been directly linked to cardiovascular disease (CVD) and may also be considered as a potential producer of short-chain fatty acids (SCFA) (Jie et al., 2017; Kim et al., 2018; Parker et al., 2020). Genus *Rikenellaceae_RC9_gut_group* was proved to promotes lipid metabolism (Zhou et al., 2018). *Unclassified_f_Ruminococcaceae* (*Ruminococcus* belongs to this family) and *Alistipes* are associated with immune regulation and healthy homeostasis and are regarded as potentially beneficial bacteria (Kong et al., 2016; Shang et al., 2016; Wang et al., 2018), Which might relate to the decrease in pro-inflammatory factors in T1 group. *Alistipes* have been shown to produce sulfolipids (SL) as a marker of a high-fat diet (Walker et al., 2017). This may explain why the growth performance of mice in the T2 group was better than that of mice in other three groups, and why the serum TG content of the mice in the T2 group was significantly higher than that of the mice in CK group. *Roseburia* belongs to the phylum Firmicutes, class Clostridia, order Clostridiales, and family Lachnospiraceae. The five well-characterized species of *Roseburia* (*Roseburia intestinalis*, *Roseburia hominis*, *Roseburia inulinivorans*, *Roseburia faecis*, and *Roseburia cecicola)* all produce SCFA, such as acetate, propionate, and butyrate (Nie et al., 2021). *Roseburia* has been shown to prevent intestinal inflammation and maintain energy homeostasis through the production of metabolites (Kumari, 2013; Rajilić-Stojanović et al., 2013). Similarly, the *Family_XIII_AD3011_ group* has probiotic properties and improves intestinal health in weaned piglets after inoculation (Yu et al., 2020). The main species that significantly increased at the genus level in the T2 group compared to the CK group were *Bacillus*, *unclassified_f_Lachnospiraceae* and *Lachnospiraceae_NK4A136_group*, and the main species that significantly decreased were *Staphylococcus* and *Aerococcus*. The genus *Bacillus* is a diverse group of gram-positive, spore-forming aerobic bacilli (Weber and Rutala, 2016). Scientists at the National Institutes of Health (NIH) found that probiotic *Bacillus* can comprehensively eradicate intestinal as well as nasal *S. aureus* colonization (Piewngam et al., 2018), this might be the reason why *Staphylococcus* significantly decreased in T2 group compared to CK group. *Lachnospiraceae_NK4A136_group* can produce SCFAs through fermentation of dietary polysaccharides and has been negatively associated with several metabolic diseases and chronic inflammation (Truax et al., 2018; Hu et al., 2019; Ma et al., 2020). *Staphylococcus aureus* is a gram-positive bacterium with a diameter of 1 μm that can cause many forms of infection (Cheung et al., 2021). *Aerococcus* are granulocyte-positive bacteria that grow in clusters, and studies have shown that *Aerococcus* are human pathogens that can cause invasive human infections, such as urogenital focal sepsis and infective endocarditis (Williams et al., 1953; Senneby et al., 2012; Senneby et al., 2014; Senneby et al., 2015; Opota et al., 2016). The improved growth performance of mice in the T3 group may be associated with a significant increase in *Christensenellaceae_R-7_group* bacteria spp. *Christensenellaceae_R-7_group* is a member of the *Christensenellaceae* family. The *Christensenellaceae* family is a relatively new family of bacteria previously associated with host health (Waters and Ley, 2019). *Christensenellaceae* have been shown to be associated with the proteolytic metabolism of god is animal proteins and intestinal metabolites are positively correlated and are strongly associated with butyrate production (Beaumont et al., 2017; Haas and Blanchard, 2017; Manor et al., 2018).

### 4.2 Relationship between the gene expression in the gut and growth performance

The productive performance of the host can be modulated by regulating the structure of the gut microflora, which in turn is largely influenced by genetics and feed. Gut microbes are also able to influence host gene expression and methylation levels (Levy et al., 2015). Anhê et al. (2019) found that altering the intestinal flora of mice can change gene expression in the gut and can prevent obesity by increasing the energy trumpet in diet-induced obese mice. Similarly, in the current study, we found significant differences in intestinal gene expression in several experimental groups of mice compared to CK group. In the T1 group, the up-regulated genes mainly included lipid metabolism-related genes such as Apoh, Apoa2, Apob, Apoa5, Apoc3, Apoa1, and Fabp1. We found these genes in the National Center for Biotechnology Information (NCBI) database to be homologous to both human and mouse. The Apoh gene was found to inhibit fatty acid oxidation and promote lipid synthesis (Pan et al., 2022). Zaki et al. (2013) found that Apoa2 is strongly associated with obesity and lipid metabolism, and that it has a potential role in regulating appetite in humans; the Apob gene is also involved in fat digestion and absorption (Honda et al., 2022). Apoa5 is a major gene involved in triglyceride metabolism (Tai and Ordovas, 2008). Apoc3 is expressed predominantly in the intestine, where it contributes to postprandial coeliac formation, and in the liver, where it regulates central lipid metabolism and turnover (Kohan, 2016). The *in vivo* association of Apoc3, Apoa1, and Apoa5 with hyperlipidemia, particularly TG levels, has been demonstrated in genome-wide association studies (GWAS) (Mar et al., 2004; Lai et al., 2005; Qi et al., 2007; Pouwer et al., 2020). This may account for the significant increase in serum TG levels in DBA mice that received fecal microbiome transplants from normal C57BL/6 mice. However, DBA mice that received fecal colony transplants from high-fat C57BL/6 mice upregulated cancer-related genes containing Mycn and C4bp (Kopylov et al., 2020; Massó-Vallés et al., 2020). This may be due to the fact that a high-fat diet enhances cancer incidence by modulating gut microbes and metabolites (Yang et al., 2022).

### 4.3 Relationship between the DEGs and bacterial community composition in the gut

Generally, intestinal bacteria interact with the expression of certain genes in intestinal epithelial cells (Larsson et al., 2012; Broderick et al., 2014; Richards et al., 2019). When human colonic epithelial cells (HCoEpiC) were treated with live gut microbiota extracted from five healthy humans, the most intense response (3,240 genes in any of the five microbiota samples) occurred at 2 h; 588 pairs of taxon-by-taxon unit transcripts corresponded to 121 host genes, and changes in expression correlated with the abundance of 46 taxa (Richards et al., 2019). Larsson et al. (2012) and Broderick et al. (2014) found that MyD88 is essential for microbiota-induced colonic expression of the antimicrobial genes Reg3β and Reg3γ in the epithelium. Lack of MyD88 resulted in altered bacterial diversity and a greater proportion of segmented filamentous bacteria in the small intestine. Su et al. found that the Aeromonas and Roseomonas percentage, as well as differential expression of IL12, were related to anti-disease ability (Su et al., 2021), and that the percentage of thick-walled bacteria was associated with growth performance of a new strain of Yellow River carp. Alenghant et al. found that a mouse model with tissue-specific deletion of histone deacetylase 3 (HDAC3) resulted in dysregulation of microbiome-dependent gene regulation in intestinal epithelial cells (Alenghat et al., 2013). Specifically, an intestinal epithelial cell-specific HDAC3 knockout mouse line (HDAC3 ΔIEC) was bred to study IBD progression. Conventionalized HDAC3 ΔIEC mice exhibit dysregulated host gene expression and disrupted homeostasis *in vivo*. In contrast, no such dysregulation was observed in the germ-free HDAC3ΔIEC mouse model, suggesting that these effects are mediated by the microbiome (Alenghat et al., 2013). From a molecular perspective, transcription factors are host proteins that bind to DNA and regulate gene transcription, and elements of the microbiome bind directly to transcription factors (Krautkramer et al., 2016; Davison et al., 2017), which may be the molecular mechanism that facilitates the linkage between microbiome and host gene expression. In this study, we analyzed the selection and CK groups in conjunction with the structure of gut bacterial communities and DEGs, and identified the Apoa2 and Apoa5 genes that were significantly associated with the *Rikenellaceae_RC9_gut_group* bacteria, which have been found to promote lipid digestion and absorption, and Apoa2 and Apoa5, on the other hand, are important genes in lipid metabolism. Thus, FMT from C57BL/6 mice might enhance the lipid metabolism of DBA mice by increasing the *Rikenellaceae_RC9_gut_group* and finally upregulated the expression of Apoa2 and Apoa5 genes in the colon.

These results suggest that gut bacteria can influence gene expression in the gut, where dominant species or bacterial structure may reflect the genetic characteristics of the host, C57BL/6 mice. Gene profiling of gut bacteria in the gut contributes to host health and performance. In this study, we used multisource FMT experimental design, in which fecal bacterial fluids derived from C57BL/6 mice, high-fat C57BL/6 mice, and Wistar rats were transplanted into DBA mice. This multisource FMT design facilitated a more comprehensive comparison of the effects of different microbial compositions on host growth and physiological functions. In addition, the effects of FMT on DBA mice were investigated by combining multilevel analyses such as 16 s rRNA sequencing and colonic gene expression sequencing. This combined multi-omics analysis helps to deeply explore the microbial regulatory mechanisms on the host, providing more precise targets for future intervention and treatment of related diseases or growth promotion. However, the mechanism of the interaction between gut flora and intestinal gene expression, as well as the mechanism of the differences between normal C57BL/6 mice and high-fat C57BL/6 mice produced as donors still need to be validated by further studies.

## 5 Conclusion

In the present study, we found that cross-strain FMT can improve the production performance of mice, which may be due to FMT altering the structure of the intestinal bacterial community in the recipient mice and subsequently enhancing lipid metabolism production, and lipid metabolites can alter intestinal gene expression in mice.

## Data availability statement

The datasets presented in this study can be found in online repositories. The names of the repository/repositories and accession number(s) can be found at: https://www.ncbi.nlm.nih.gov/sra/PRJNA1050037, NCBI-PRJNA1050037.

## Ethics statement

The animal study was approved by Animal Care Advisory Committee of Zhejiang University, Hangzhou, China. The study was conducted in accordance with the local legislation and institutional requirements.

## Author contributions

WY: Conceptualization, Formal analysis, Investigation, Methodology, Supervision, Visualization, Writing – original draft, Writing – review & editing. JF: Conceptualization, Visualization, Writing – review & editing. WW: Formal analysis, Writing – original draft. ZC: Investigation, Writing – original draft. QH: Methodology, Writing – original draft. LQ: Supervision, Visualization, Writing – review & editing.

## Footnotes

^1^ https://cloud.majorbio.com
