# Supplementary material for: Effects of fecal microbiota transplantation on metabolic health of DBA mice
Source: Front Microbiol. 2024 Feb 20;15:1352555. doi: 10.3389/fmicb.2024.1352555 (PMC10912182; doi:10.3389/fmicb.2024.1352555)
Supplement: Supplementary file 2 [file Data_Sheet_1.docx]

**Supplementary Table 1.** Effect of FMT on growth performance of DBA mice

| **Items** | **Treatment** | | | | **SEM** | ***P-*Value** |
| --- | --- | --- | --- | --- | --- | --- |
|  | **T1** | **T2** | **T3** | **CK** |  |  |
| IBW (g) | 15.77 ± 0.44 | 15.44 ± 0.46 | 15.34 ± 0.38 | 15.01 ± 0.31 | 0.19 | 0.649 |
| FBW (g) | 21.85^b^ ± 0.34 | 22.96^a^ ± 0.22 | 21.89^b^ ± 0.31 | 20.76^c^ ± 0.48 | 0.21 | 0.003^**^ |
| ADG (g/d) | 0.22^b^ ± 0.01 | 0.27^a^ ± 0.01 | 0.25^ab^ ± 0.01 | 0.20^bc^ ± 0.01 | 0.01 | 0.001^**^ |

^a-b^ Means with different superscripts in the same row indicate significantly difference (*p* < 0.05). IBW: initial body weight; FBW: final body weight; ADG: average daily gain; CK: DBA mice accepting saline gavage; T1: DBA mice receiving fecal transplants from C57BL/6 mice; T2: DBA mice receiving fecal transplants from high-fat C57BL/6 mice; T3: DBA mice receiving fecal transplants from Wistar rats; SEM: standard error means.

**Supplementary Table 2.** The effects of FMT on immune function in DBA mice

| **Items (ng/L)** | **Treatment** | | | | **SEM** | ***P-*Value** |
| --- | --- | --- | --- | --- | --- | --- |
|  | **T1** | **T2** | **T3** | **CK** |  |  |
| CXCL1 | 13.04 ± 1.38 | 13.02 ± 0.78 | 12.74 ± 2.01 | 15.62 ± 2.58 | 1.02 | 0.709 |
| CXCL2 | 38.55^b^ ± 2.26 | 48.33^a^ ± 2.26 | 47.98^a^ ± 2.61 | 55.38^a^ ± 3.72 | 1.68 | 0.005^**^ |
| TNF-α | 324.18^a^ ± 18.07 | 291.00^b^ ± 13.27 | 329.64^a^ ± 22.13 | 355.44^a^ ± 13.00 | 9.17 | 0.018^*^ |
| IL-1β | 54.71 ± 3.64 | 56.91 ± 3.23 | 59.14 ± 3.29 | 59.06 ± 1.63 | 1.46 | 0.693 |
| IL-6 | 71.24 ± 4.14 | 79.29 ± 2.36 | 70.45 ± 6.28 | 77.05 ± 3.77 | 2.07 | 0.342 |
| IL-22 | 64.57^bc^ ± 2.70 | 71.95^ab^ ± 4.53 | 69.60^b^ ± 4.06 | 82.29^a^ ± 3.97 | 2.13 | 0.024^*^ |
| IL-10 | 833.57 ± 29.47 | 817.50 ± 32.08 | 785.77 ± 28.14 | 854.55 ± 42.86 | 17.09 | 0.515 |
| IgA | 0.38 ^b^ ± 0.03 | 0.57 ^b^ ± 0.08 | 0.70^a^ ± 0.05 | 0.49^b^ ± 0.17 | 0.03 | 0.001^**^ |

^a-c^ Means with different superscripts in the same row indicate significantly difference (*p* < 0.05). CXCL1: chemokine (C-X-C motif) ligand 1; CXCL2: chemokine (C-X-C motif) ligand 2; IgA: immunoglobulin A; TNF-α:Tumor Necrosis Factor-α; IL-1β: Interleukin-1β; IL-6: Interleukin 6; IL-22: Interleukin 22; IL-10: Interleukin 10; CK: DBA mice accepting saline gavage; T1: DBA mice receiving fecal transplants from C57BL/6 mice; T2: DBA mice receiving fecal transplants from high-fat C57BL/6 mice; T3: DBA mice receiving fecal transplants from Wistar rats; SEM: standard error means.

**Supplementary Table 3.** The effects of FMT on lipids, liver function and antioxidant function in DBA mice

| **Items** | **Treatment** | | | | **SEM** | ***P-*Value** |
| --- | --- | --- | --- | --- | --- | --- |
|  | **T1** | **T2** | **T3** | **CK** |  |  |
| TG (mmol/L) | 2.00^a^ ± 0.16 | 1.50^b^ ± 0.20 | 1.05 ^c^ ± 0.34 | 0.93^c^ ± 0.12 | 0.10 | ＜0.001^**^ |
| TC (mmol/L) | 1.96 ± 0.36 | 2.05 ± 0.28 | 2.42 ± 0.49 | 1.91 ± 0.22 | 0.17 | 0.758 |
| TP (g/L) | 26.17 ± 1.74 | 25.15 ± 2.55 | 30.16 ± 2.90 | 24.17 ± 15.89 | 1.81 | 0.746 |
| ALB (g/L) | 22.12 ± 1.82 | 22.92 ± 2.20 | 19.07 ± 2.50 | 20.54 ± 2.00 | 1.05 | 0.614 |
| SOD (U/mL) | 21.87 ± 0.16 | 22.43 ± 0.90 | 22.47 ± 0.69 | 22.05 ± 1.12 | 0.42 | 0.963 |

^a-c^ Means with different superscripts in the same row indicate significantly difference (*p* < 0.05). TG: Triglyceride; TC: Total cholesterol; ALB: Albumin; TP: Total protein; SOD: Serum Superoxide Dismutase; CK: DBA mice accepting saline gavage; T1: DBA mice receiving fecal transplants from C57BL/6 mice; T2: DBA mice receiving fecal transplants from high-fat C57BL/6 mice; T3: DBA mice receiving fecal transplants from Wistar rats; SEM: standard error means.

**Table 4.** Relationship between differentially expressed genes and bacteria flora on genus level between T1 and CK

|  | Fgb | Apoa2 | Apoa5 | Dgat2 | Cyp2c29 | Plg | C8a | C8b | Angptl4 |
| --- | --- | --- | --- | --- | --- | --- | --- | --- | --- |
| Alistipes | 0.561 | 0.359 | 0.668 | 0.078 | 0.135 | 0.667 | 0.664 | 0.217 | 0.055 |
| Rikenellaceae_RC9_gut_group | 0.016* | 0.001** | 0.005** | 0.203 | 0.010* | 0.030* | 0.065 | 0.004** | 0.407 |
| unclassified_f__Ruminococcaceae | 0.044* | 0.079 | 0.655 | 0.105 | 0.003** | 0.114 | 0.039* | 0.012 | 0.087 |
| Roseburia | 0.271 | 0.207 | 0.724 | 0.047* | 0.044* | 0.363 | 0.293 | 0.088 | 0.039* |
| Family_XIII_AD3011_group | 0.642 | 0.439 | 0.941 | 0.112 | 0.207 | 0.536 | 0.630 | 0.287 | 0.219 |

*: p≤0.05, **: p≤0.01CK: DBA mice accepting saline gavage; T1: DBA mice receiving FMT from C57BL/6 mice.

**Table 5.** Relationship between differentially expressed genes and bacteria flora on genus level between T2 and CK

|  | Angptl8 | Cyp8b1 | C4bp | Bmp8a | Fanca |
| --- | --- | --- | --- | --- | --- |
| Bacillus | 0.034* | 0.011* | 0.025* | 0.070 | 0.544 |
| Staphylococcus | 0.007** | 0.074 | 0.013* | 0.001** | 0.045* |
| unclassified_f__Lachnospiraceae | 0.016* | 0.064 | 0.002** | 0.104 | 0.152 |
| Lachnospiraceae_NK4A136_group | 0.020* | 0.192 | 0.093 | 0.002** | 0.015* |
| Aerococcus | 0.001** | 0.003** | 0.034* | 0.018* | 0.026* |
| Roseburia | 0.036* | 0.225 | 0.136 | 0.004** | 0.005** |
| Rikenellaceae_RC9_gut_group | 0.006** | 0.001** | 0.028* | 0.086 | 0.273 |
| norank_f__Ruminococcaceae | 0.044* | 0.039* | 0.183 | 0.009** | 0.021* |
| Eubacterium_xylanophilum_group | 0.010* | 0.001** | 0.041* | 0.045* | 0.057 |
| Anaerotruncus | 0.001** | 0.051 | 0.018* | 0.021* | 0.070 |
| unclassified_f__Ruminococcaceae | 0.001** | 0.048* | 0.032* | 0.097 | 0.019* |
| norank_f__Erysipelotrichaceae | 0.005** | 0.033* | 0.234 | 0.049* | 0.093 |
| norank_f__Peptococcaceae | 0.025* | 0.086 | 0.044* | 0.001** | 0.130 |

*: p≤0.05, **: p≤0.01CK: DBA mice accepting saline gavage; T2: DBA mice receiving FMT from high-fat C57BL/6 mice.
